# An external validation of the QCOVID3 risk prediction algorithm for risk of hospitalisation and death from COVID-19: An observational, prospective cohort study of 1.66m vaccinated adults in Wales, UK

**DOI:** 10.1371/journal.pone.0285979

**Published:** 2023-05-18

**Authors:** Jane Lyons, Vahé Nafilyan, Ashley Akbari, Stuart Bedston, Ewen Harrison, Andrew Hayward, Julia Hippisley-Cox, Frank Kee, Kamlesh Khunti, Shamim Rahman, Aziz Sheikh, Fatemeh Torabi, Ronan A. Lyons

**Affiliations:** 1 Faculty of Medicine, Health & Life Science, Population Data Science, Swansea University Medical School, Swansea University, Swansea, United Kingdom; 2 Office of National Statistics, Newport, United Kingdom; 3 Usher Institute, Centre for Medical Informatics, University of Edinburgh, Edinburgh, United Kingdom; 4 Department of Epidemiology and Public Health, University College London, London, United Kingdom; 5 Nuffield Department, Primary Care Health Sciences, University of Oxford, Oxford, United Kingdom; 6 School of Medicine, Dentistry and Biomedical Sciences, Queen’s University Belfast, Belfast, United Kingdom; 7 Diabetes Research Centre, University of Leicester, Leicester, United Kingdom; 8 Department of Health and Social Care, Mental Health and Disabilities Analysis, London, United Kingdom; 9 Usher Institute, University of Edinburgh, Edinburgh, United Kingdom; Elazıg Fethi Sekin City Hospital: Elazig Fethi Sekin Sehir Hastanesi, TURKEY

## Abstract

**Introduction:**

At the start of the COVID-19 pandemic there was an urgent need to identify individuals at highest risk of severe outcomes, such as hospitalisation and death following infection. The QCOVID risk prediction algorithms emerged as key tools in facilitating this which were further developed during the second wave of the COVID-19 pandemic to identify groups of people at highest risk of severe COVID-19 related outcomes following one or two doses of vaccine.

**Objectives:**

To externally validate the QCOVID3 algorithm based on primary and secondary care records for Wales, UK.

**Methods:**

We conducted an observational, prospective cohort based on electronic health care records for 1.66m vaccinated adults living in Wales on 8^th^ December 2020, with follow-up until 15^th^ June 2021. Follow-up started from day 14 post vaccination to allow the full effect of the vaccine.

**Results:**

The scores produced by the QCOVID3 risk algorithm showed high levels of discrimination for both COVID-19 related deaths and hospital admissions and good calibration (Harrell C statistic: ≥ 0.828).

**Conclusion:**

This validation of the updated QCOVID3 risk algorithms in the adult vaccinated Welsh population has shown that the algorithms are valid for use in the Welsh population, and applicable on a population independent of the original study, which has not been previously reported. This study provides further evidence that the QCOVID algorithms can help inform public health risk management on the ongoing surveillance and intervention to manage COVID-19 related risks.

## Introduction

Following the emergence of the SARS-CoV-2 infection and at the start of the COVID-19 pandemic, there was an urgent public health need to identify individuals at highest risk of severe outcomes, in particular hospitalisation and death following infection. To support the National Health Service (NHS) and protect the most clinically vulnerable individuals, the Chief Medical Officer for England commissioned the New and Emerging Respiratory Virus Threats Advisory Group (NERVTAG), an expert committee of the Department of Health and Social Care who advise the UK government, to develop the QCOVID risk assessment algorithms for predicting risk of COVID-19 related hospital admissions or death [1]. The algorithms were developed on individual demographic and clinical characteristics from six million primary care patients registered at 1,205 English general practices. Performance metrics demonstrated the predictive algorithms had high levels of discrimination and were well calibrated, which was also shown in three independent validation studies [2–4]. The calculated risk scores from these algorithms saw an additional ~1.5 million people added to the national shielding patient list, and ~800,000 of those prioritised for vaccination if they had not already received it, highlighting the importance and need for these population risk prediction algorithms for planning and patient management in the case of future infection spikes and pandemics [5]. These original QCOVID algorithms were developed using data from the first wave of the COVID-19 pandemic, prior to the national rollout of the vaccination programme.

Despite the success and effectiveness of the vaccine programme, the discovery of new variants alongside studies showing waning of immunity over time has demonstrated that there remains a risk of COVID-19 infection and subsequent COVID-19 related hospitalisation and death following vaccination [6–9]. The vaccines efficacy were tested generally on younger healthier volunteers in clinical trials [10]. It is important to identify risk factors associated with COVID-19-related hospitalisation and deaths in all individuals following vaccination since not all patients will achieve immunity. The UK Government advisory group NERVTAG further developed the QCOVID3 risk assessment algorithms to identify groups of people at highest risk of severe COVID-19 related outcomes following one or two doses of vaccine [11]. The QCOVID3 risk algorithms were developed on data from the second wave of the pandemic in England, UK, and includes some additional predictor variables such as vaccine dose (first or second), extending the categorisation of severity of diabetes to include glycated haemoglobin levels, bipolar disorder, schizophrenia, and a seven-day moving average of the background rates of positive SARS-CoV-2 tests per 100,000 people to account for changing infection rates. The risk scores from these algorithms provide further evidence to prioritise high-risk individuals who may need further interventions, such as additional booster vaccinations or treatment with monoclonal antibodies, antivirals or pre-exposure prophylaxis (Evushield) [12]. This was designed to help protect high-risk individuals with the ending of social distance measures, mandatory testing, and self-isolation in the UK.

It is important to replicate and validate prediction algorithms in independent populations to ensure they work in an ‘out of sample’ setting particularly if they could be used clinically in this setting. It is also to inform policy development at a national scale and contribute to the planning and management of individual patient care as well as contribute to the planning of the prevention of future pandemics [13,14]. Pandemic predictive risk assessment algorithms, such as QCOVID3, can be used to identify vulnerable groups of individuals at the highest risk of serious health outcomes as well as identify demographic and clinical groups of individuals who are more or less likely to partake in the uptake of a preventative intervention during a pandemic. Outputs from validating these prediction algorithms can be used to highlight vulnerabilities within healthcare systems as well identify variations in service provision and uptake of vaccinations for planning and managing patient care for future pandemics.

Validation studies were funded to compare the performance of the updated algorithms in each of the four nations in the UK to ensure external validity and provide evidence on the application of the algorithms in managing patient risk over time in different populations [15]. The aim of this particular study was to independently validate the updated published QCOVID3 risk prediction algorithms for risk of COVID-19-related deaths and hospitalisation in vaccinated adults having one or two doses of vaccination by 15^th^ June 2021 in Wales, UK.

## Materials and methods

### Study design

We conducted an observational, longitudinal, cohort study of vaccinated adults living in Wales from 8^th^ December 2020, with follow-up until 15^th^ June 2021. The outcomes of interest were time to COVID-19 related death and hospitalisation. We assessed the performance of the QCOVID3 algorithms using measures of discrimination and calibration. This paper mirrors the published English study and follows the STROBE and TRIPOD reporting guidelines [11,16,17].

### Data sources

This study used routinely collected anonymised, individual-level, population-scale health and demographic data held in the Secure Anonymised Information Linkage (SAIL) Databank to create a retrospective population-based individual-level linked e-cohort [18,19]. For this analysis, we used the Welsh Demographic Service Dataset (WDSD), Welsh Longitudinal General Practice (WLGP), Annual District Death Extract (ADDE) from the Office for National Statistics (ONS) mortality data, Annual District Death Daily (ADDD), Consolidated Death Data Source (CDDS), COVID Vaccination Dataset (CVVD), Patient Episode Database for Wales (PEDW), Care Homes Index (CARE), COVID-19 Test Results (PATD), 2011 Census Wales (CENW), and UK Government published daily infection rates data [20,21].

### Sample inclusion criteria and follow up

We defined the population of interest as vaccinated adults living in Wales on 8^th^ December 2020 with follow-up until 15^th^ June 2021. Individuals included were aged between 19 and 100 on the 8^th^ December 2020, registered with a SAIL-providing general practice (86% of Welsh general practices), and who had received one or two vaccinations of Oxford-AstraZeneca or Pfizer-BioNTech within the study period (S1 Fig). Follow-up started from 14 days after receiving each vaccine dose until they had the outcome of interest (COVID-19-related death or hospitalisation), died, migrated out of Wales, or until the end of the study period. Individuals who were vaccinated within 14 days of the study end date were not included due to insufficient follow up time. Individuals who only received one dose of the vaccine during the study period were followed up from 14 days post vaccination until the outcome of interest, death, migration out of Wales, or until the end of the study period. Individuals who received two doses of vaccination were, followed up over two time periods. For the first period, individuals were followed up from 14 days post first vaccination until 14 days after their second vaccination. For the second period, individuals were followed up from 14 days post second vaccination until outcome of interest, death, migration out of Wales, or until the end of the study period.

### Outcome of interest

The primary and secondary outcomes were COVID-19-related death and hospitalisation respectively, with time-at-risk calculated from 14 days after vaccination. We utilised a combination of ADDE, ADDD, WDSD and CDDS to identify all deaths of Welsh residents, inclusive of in-hospital and out of hospital deaths. Deaths involving COVID-19 were identified using the tenth revision of the International Classification of Diseases (ICD-10) codes U07.1 or U07.2, or from text fields containing the causes of death within the data sources (ADDD, CDDS). Additionally, deaths involving COVID-19 were also included if the death occurred within 28 days of a positive SARS-CoV-2 infection using the PATD data.

COVID-19-related hospital admission were included if they contained U07.1 or U07.2 ICD10 codes, or, any emergency admission within 14 days following a positive polymerase chain reaction (RT-PCR) COVID-19 test result. Individuals who had a COVID-19 hospitalisation prior to the study start date were not included in the hospital analysis.

### Predictor variables

Predictive demographic, clinical, and pharmaceutical variables (Box 1) to validate the updated algorithms were based on the original QCOVID studies [1–4], which includes the clinical vulnerability group criteria used to identify those advised to shield at the start of the pandemic and risk factors associated with adverse outcomes for respiratory diseases [22,23].

**Box 1. List of predictor variables for the QCOVID3 risk equations for vaccinated individuals**

**Demographic**

    • Age in years on 8^th^ December 2020

    • Biological sex at birth

    • Townsend Deprivation Score

    • Ethnicity

    • What is your housing category—care home, homeless or neither?

Have you had a 1^st^ or 2^nd^ dose of Oxford-AstraZeneca or Pfizer-BioNTech COVID-19 vaccination?

    • What is the background daily rate per 100,000 for SARS-CoV-2 infection in the last 7 days?

**Lifestyle**

Body Mass Index

**Conditions**

    • Have you had chemotherapy in the last 12 months?

    • Have you had radiotherapy in the last 6 months?

    • Do you have sickle cell disease?

    • Have you a cancer of the blood or bone marrow such as leukaemia, myelodysplastic syndromes, lymphoma or myeloma and are at any stage of treatment?

    • Do you have lung or oral cancer?

Do you have a learning disability or Down’s syndrome?

    • Do you have Chronic Kidney Disease (CKD) and at what stage?

    • Do you have diabetes?

    • Do you have Parkinson’s disease?

    • Do you have epilepsy?

    • Do you have dementia?

    • Do you have Chronic Obstructive Pulmonary Disease (COPD)?

    • Do you have motor neurone disease, multiple sclerosis, myasthenia, or Huntington’s chorea?

    • Do you have coronary heart disease?

    • Do you have heart failure?

    • Do you have peripheral vascular disease?

    • Do you have atrial fibrillation or atrial flutter?

    • Do you have cirrhosis of the liver?

    • Have you had a thrombosis or pulmonary embolus?

Have you had a stroke or transient ischaemic attack?

Do you have bipolar disease or schizophrenia?

Do you have severe combined immunodeficiency?

    • Have you had a solid organ transplant ever or bone marrow transplant in the last 6 months?

For the demographic variables, the WDSD was used to define age, sex, and Townsend score. Townsend score is a measure of deprivation, based on the area of residence, with a higher score indicating a higher level of deprivation [24]. The 2011 Census Wales (CENW) is linked to the cohort in order to derive ethnic groups (i.e. Bangladeshi, Black African, Black Caribbean, Chinese, Indian, Pakistani, Mixed, Other, and White). The ethnic group variable also had a category corresponding to ‘not recorded/unknown’. This category was used whenever the corresponding value was missing (Table 1). To adjust for changing infection rates over the study period, a seven-day moving average of the background rates of positive SARS-CoV-2 tests per 100,000 people using published data was added to the algorithms [20].

**Table 1:** Demographic and clinical characteristics for the total cohort and those who died or were admitted to hospital with COVID-19.

	Overall		COVID-19 deaths		COVID-19 admissions	
	N	%	N	%	N	%
**Overall**	1,656,154		353		744	
Male	787,878	47.57	178	50.42	332	44.62
Female	868,276	52.43	175	49.58	412	55.38
Mean age in years (SD)	53.92 (17.99)		81.23 (10.60)		75.01 (15.18)	
Mean SARS-CoV-2 daily infection rate per 100,000 population (SD)	21.70 (27.78)		30.0 (17.2)		25.1 (19.3)	
**Age group, years**						
19–29	184,667	11.15	0	0.00	0	0.00
30–39	225,672	13.63	^		22	2.96
40–49	258,118	15.59	^		41	5.51
50–59	327,717	19.79	^		59	7.93
60–69	286,761	17.31	46^	13.03	84	11.29
70–79	242,476	14.64	76	21.53	176	23.66
80–89	109,645	6.62	160	45.33	262	35.22
≥90	21,098	1.27	71	20.11	100	13.44
COVID-19 vaccine 1 dose only	736,113	44.45	333	94.33	628	84.41
COVID-19 vaccine 2 doses	920,041	55.55	20	5.67	116	15.59
**Ethnic group**						
Bangladeshi^	10,617	0.64	20+	5.67+	38	5.11
Black African	5,130	0.31	^		^	
Black Caribbean	1,636	0.10	^		^	
Chinese	4,595	0.28	^		^	
Indian	8,235	0.50	^		^	
Mixed	9,975	0.60	^		^	
Other	20,084	1.21	^		^	
Pakistani	6,056	0.37	^		^	
White	1,575,332	95.12	320	90.65	686	92.20
Not recorded	14,494	0.88	^		^	
**Townsend deprivation quintile**						
1 (most affluent)	301,180	18.19	49	13.88	99	13.31
2	365,881	22.09	83	23.51	165	22.18
3	483,814	29.21	119	33.71	236	31.72
4	364,976	22.04	77	21.81	177	23.79
5 (most deprived)	140,303	8.47	25	7.08	67	9.01
**Accommodation**						
Neither homeless nor care home	1,642,816	99.19	288	81.59	686	92.20
Care home or homeless	13,338	0.81	65	18.41	58	7.80
**Body mass index, kg/m** ^**2**^						
<18.5	15,736	0.95	25	7.08	18	2.42
18.5 to <25	249,265	15.05	76	21.53	151	20.30
25 to <30	328,900	19.86	84	23.80	174	23.39
≥30	373,628	22.56	79	22.38	216	29.03
BMI not recorded	688,625	41.58	89	25.21	185	24.87
**Chronic Kidney Disease**						
No Chronic Kidney Disease	1,577,939	95.28	245	69.41	558	75.00
Stage 3	69,109	4.17	81	22.95	137	18.41
Stage 4	3,657	0.22	16	4.53	20	2.69
Stage 5	5,449	0.33	11	3.12	29	3.90
**Learning disability**						
No learning disability	1,632,181	98.55	343+	97.17+	729	97.98
Learning disability	23,735	1.43	^		15	2.02
Down Syndrome	238	0.01	^		0	0.00
**Cancer and immunosuppression**						
No chemotherapy in past 12 months	1,649,881	99.62	337	95.47	724	97.31
Chemotherapy in past 12 months	6,273	0.38	16	4.53	20	2.69
Blood cancer	10,541	0.64	*		19	2.55
Respiratory cancer	5,711	0.34	10	2.83	12	1.61
Radiotherapy in past 6 months	1,560	0.09	*		*	
Solid organ transplant ever or bone marrow transplant in past 6 months	866	0.05	*		0	0.00
Dispensed immunosuppressant medication	3038	0.18	*		*	
Dispensed leukotriene or LABA	81251	4.91	37	10.48	89	11.96
Dispensed regular prednisolone	15283	0.92	15	4.25	43	5.78
**Other pre-existing conditions**						
Diabetes						
No diabetes	1,495,453	90.30	237	67.14	551	74.06
Type 1 diabetes	7,663	0.46	^		^	
Type 2 diabetes	153,038	9.24	105+		182+	
COPD	64,002	3.86	54	15.30	112	15.05
Coronary heart disease	87,802	5.30	90	25.50	142	19.09
Stroke	54,611	3.30	66	18.70	107	14.38
Atrial fibrillation	62,640	3.78	95	26.91	133	17.88
Heart failure	31,135	1.88	54	15.30	83	11.16
Venous thromboembolism	43,523	2.63	39	11.05	81	10.89
Peripheral vascular disease	17,736	1.07	26	7.37	34	4.57
Dementia	17,166	1.04	77	21.81	74	9.95
Parkinson’s disease	5,528	0.33	16	4.53	17	2.28
Epilepsy	23,990	1.45	*		20	2.69
Rare neurological conditions	5,664	0.34	*		*	
Bipolar disease or schizophrenia	13,712	0.83	*		14	1.88
Severe combined immunodeficiency	723	0.04	0	0.00	*	
Cirrhosis of the liver	7,275	0.44	*		14	1.88
Sickle cell disease	55	0.00	0	0.00	0	0.00

^ Aggregation of figures and masking of zero.

* Less than 10 have been masked.

The majority of pre-existing conditions were identified in the WLGP primary care data source using Read codes version 2 (CTV2). Where no timeframe was stated, a lookback period was used from 1^st^ January 1998 to the study start date (14 days after first vaccination). For body mass index (BMI), the latest BMI measurement within 5 years was used. BMI records outside this time period and BMIs <15 and >47 were set to missing, with the mean BMI replacing missing values. The highest BMI was included if an individual had multiple BMI records on the latest date. For diabetes, if the latest health record had defined an individual with both type 1 and type 2 diabetes, type 2 took precedence. Patients with diabetes were further categorised by severity according to the most recent HBA1C level in their primary care records (HBA1C levels were categorised at a threshold of 59 mmol/mol). For the housing covariate, if the latest record defined an individual as being homeless and living in a care home, then living in a care home took precedence. For the learning disabilities covariate, if the latest record identified an individual with learning disabilities and Down’s syndrome, then Down’s syndrome was prioritised.

Office of Population Censuses and Surveys (OPCS) Classification of Interventions and Procedures version 4 (OPCS-4) coded conditions in the inpatient (PEDW) data were used to identify chemotherapy status, Chronic Kidney Disease (CKD) stages, bone marrow or stem cell transplant, radiotherapy, and solid organ transplant.

### Algorithm validation

The original study developed risk models using cause specific Cox proportional hazard models to calculate hazard ratios and develop the risk scores accounting for the competing risk of death due to other causes [11]. The published QCOVID3 risk equations were applied to the cohort to calculate the risk scores for COVID-19 related hospitalisation and death respectively [25]. The following modifications for the Welsh cohort were required due to SAIL policy and data availability: HIV status was not included in the analysis. Those receiving chemotherapy within one year of study start (14 days following first vaccination) were assigned the chemotherapy group B (middle severity group) coefficients. Additionally, missing published death and vaccine times were replaced with zero [25].

Performance metrics were calculated to validate the QCOVID3 predicted risk of COVID-19 related hospitalisation and death. R^2^ values, D statistic, and Harrell’s C statistic with corresponding 95% intervals were calculated for the total cohort and by age, sex, and vaccination number [26–28]. The R^2^ values refer to the proportion of variation in survival time explained by the model. The D statistic and Harrell’s C statistic are discrimination measures that quantify the separation in survival between patients with different levels of predicted risks and the extent to which people with higher risk scores have earlier events respectively. To measure calibration, we compared the mean predicted risks with observed risks, by 20ths of predicted risk.

### Ethics statement

The use of de-identified data in SAIL complies with National Research Ethics Service (NRES) guidance. Applications to use data held within the SAIL Databank, an ISO: 27001 and UK Statistics Authority (UKSA) Digital Economy Act (DEA) accredited Trusted Research Environment, must first be approved by the independent Information Governance Review Panel (IGRP). The IGRP contains a multidisciplinary professional group, including members of the public, and it gives careful consideration to each project to ensure proper and appropriate use of SAIL data. When access has been granted, it is gained through a privacy protecting safe haven and remote access system referred to as the SAIL Gateway. SAIL project 0911 was approved by IGRP on 26^th^ June 2019 with further amendments to the scope approved to allow rapid analysis as the COVID-19 pandemic unfolded.

SAIL has established an application process to be followed by anyone who would like to access data via SAIL at https://www.saildatabank.com/application-process. Participant consent was not required for this study as all data is anonymised and further encrypted.

## Results

The study included 1,656,154 individuals (Table 1). Of these, 787,878 (47.6%) were male, the mean age was 53.9 (SD 18), 920,041 (55.6%) had received two doses of the vaccine with the median time between 1^st^ and 2^nd^ dose being 71 days (IQR: 47–77), and the majority of individuals were from White ethnic backgrounds (1,575,332, 95.1%). Overall, 991,158 (59.8%) had at least one dose of the Oxford-AstraZeneca vaccine and 665,360 (40.2%) had at least one dose of the Pfizer-BioNTech vaccine. Median follow-up time was 60 days (interquartile range 41–76) after the first dose and 48 (22–77) days after the second dose.

In total, there were 353 (0.02%) COVID-19 related deaths and 744 (0.05%) COVID-19-related hospital admissions. In general, individuals who died from COVID-19 were more likely to be male (178, 50.4%), aged 80 years and older (231, 65.4%), and living in more deprived areas (221, 62.6% in quintiles 3–5). Amongst those with a recorded BMI, 61.7% of people who died were overweight or obese. Atrial fibrillation, coronary heart disease, diabetes, and dementia were the pre-existing conditions with the highest proportions of deaths (Table 1).

Individuals with a COVID-19-related admission were more likely to be female (412, 55.4%), aged 70 years and older (538, 72.3%), and living in more deprived areas (480, 64.5% in quintiles 3–5). CKD, coronary heart disease, diabetes, and atrial fibrillation were the pre-existing conditions with the highest proportions of hospitalisations (Table 1).

Table 2 shows the performance metrics of the QCOVID3 algorithm in the Welsh cohort. The metrics have been provided for the total cohort and by age, sex, and the number of vaccinations. For COVID-19 related deaths, the algorithm explained 72.5% (95% CI: 70.3–74.4) of the variation in time to death, the Harrell’s C statistic was 0.939 (95% CI: 0.928–0.950) and the D statistic 3.321 (95% CI: 3.148–3.493). Results when restricted to individuals who only received one vaccination were 72.8% (95% CI: 69.7–75.5), 0.992 (95% CI: 0.987–0.996) and 4.796 (95% CI: 4.608–4.983) respectively. Similar results were found in males and females. Individuals who received two vaccinations and results for some age groups yielded slightly poorer metrics, which was likely due to fewer events.

**Table 2:** Performance of the QCOVID3 algorithm to predict risk of COVID-19 related death and hospitalisation for the total cohort and by age, sex, and vaccination dose (95% CI).

	COVID-19 related death	COVID-19 related admission
**Overall**		
R^2^ statistic	0.725 (0.703 to 0.744)	0.551 (0.524 to 0.576)
D statistic	3.321 (3.148 to 3.493)	2.266 (2.149 to 2.384)
Harrell’s C statistic	0.939 (0.928 to 0.950)	0.828 (0.812 to 0.845)
**Female**		
R^2^ statistic	0.728 (0.697 to 0.755)	0.545 (0.509 to 0.579)
D statistic	3.351 (3.106 to 3.596)	2.241 (2.084 to 2.399)
Harrell’s C statistic	0.943 (0.928 to 0.958)	0.826 (0.803 to 0.849)
**Male**		
R^2^ statistic	0.721 (0.689 to 0.749)	0.561 (0.521 to 0.597)
D statistic	3.291 (3.047 to 3.534)	2.313 (2.136 to 2.489)
Harrell’s C statistic	0.935 (0.918 to 0.952)	0.832 (0.808 to 0.856)
**One dose of the vaccine only**		
R^2^ statistic	0.846 (0.835 to 0.856)	0.815 (0.798 to 0.830)
D statistic	4.796 (4.608 to 4.983)	4.291 (4.066 to 4.516)
Harrell’s C statistic	0.992 (0.987 to 0.996)	0.939 (0.914 to 0.963)
**Two doses of vaccine**		
R^2^ statistic	0.547 (0.355 to 0.679)	0.376 (0.281 to 0.463)
D statistic	2.247 (1.519 to 2.974)	1.590 (1.281 to 1.900)
Harrell’s C statistic	0.875 (0.812 to 0.938)	0.741 (0.693 to 0.789)
**Age groups**		
**19–59**		
R^2^ statistic	0.587 (0.385 to 0.717)	0.362 (0.270 to 0.447)
D statistic	2.438 (1.620 to 3.255)	1.543 (1.245 to 1.840)
Harrell’s C statistic	0.859 (0.771 to 0.948)	0.737 (0.686 to 0.788)
**60–69**		
R^2^ statistic	0.720 (0.634 to 0.781)	0.439 (0.334 to 0.529)
D statistic	3.281 (2.696 to 3.866)	1.809 (1.449 to 2.169)
Harrell’s C statistic	0.928 (0.896 to 0.961)	0.741 (0.680 to 0.802)
**70–79**		
R^2^ statistic	0.604 (0.530 to 0.666)	0.429 (0.361 to 0.491)
D statistic	2.530 (2.171 to 2.889)	1.773 (1.538 to 2.009)
Harrell’s C statistic	0.884 (0.848 to 0.919)	0.779 (0.745 to 0.813)
**80+**		
R^2^ statistic	0.557 (0.508 to 0.600)	0.358 (0.306 to 0.408)
D statistic	2.294 (2.082 to 2.506)	1.528 (1.359 to 1.697)
Harrell’s C statistic	0.856 (0.831 to 0.880)	0.752 (0.727 to 0.778)

For COVID-19 related hospital admissions, the algorithm explained 55.1% (95% CI: 52.4–57.6) of the variation in time to death, the Harrell’s C statistic was 0.828 (95% CI: 0.812–0.845) and the D statistic 2.266 (95% CI: 2.149–2.384). Results restricted to individuals who only received one vaccination yielded the highest performance with 81.5% (95% CI: 79.8–83.0), 0.939 (95% CI: 0.914–0.963) and 4.291 (95% CI: 4.066–4.516) respectively. Similar to the death outcomes, metrics for hospitalisations were not as good for those receiving two vaccinations or in sub-analyses by age groups.

**Fig 1:**
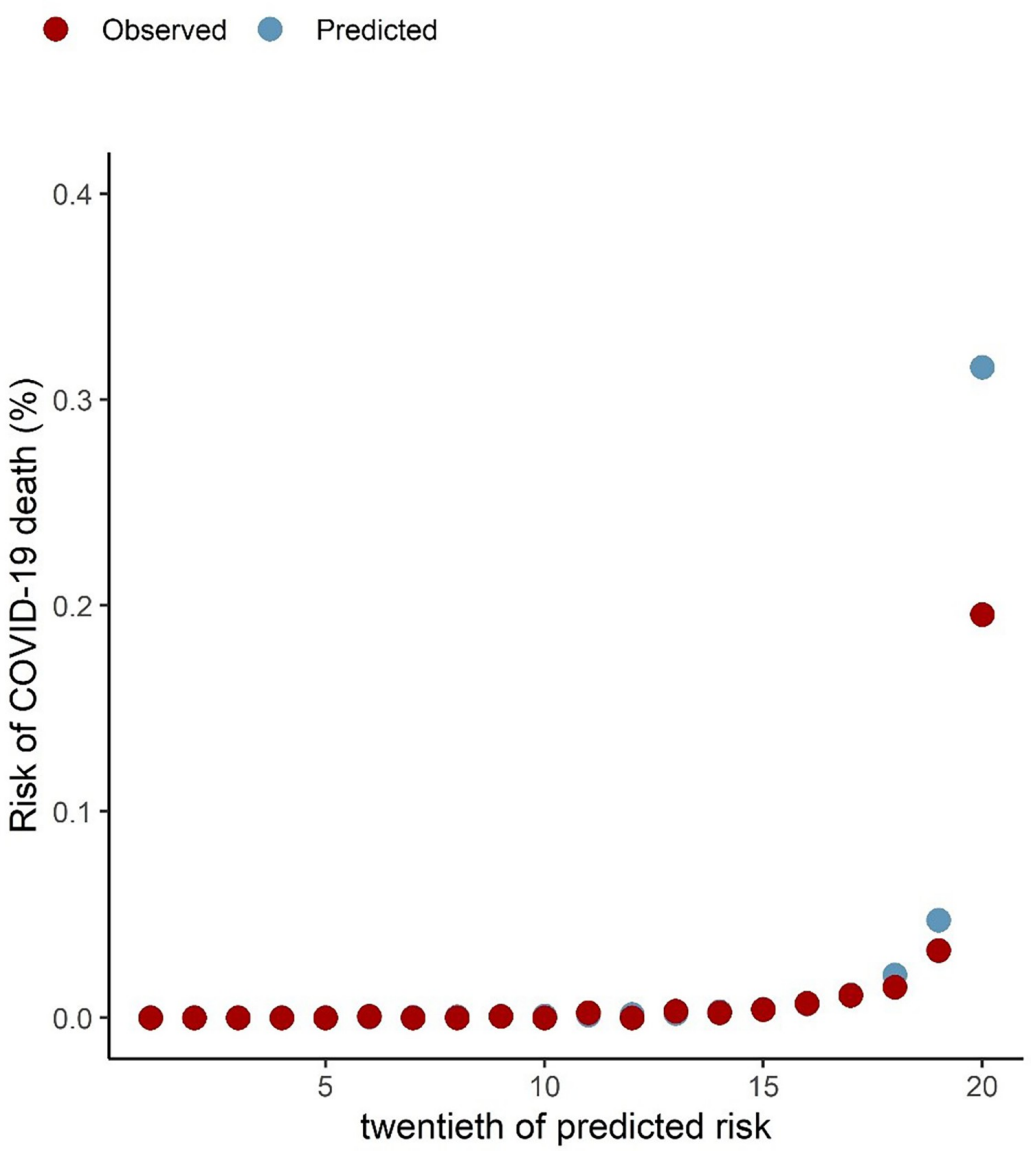
Predicted and observed risk of COVID-19 related deaths.

**Fig 2:**
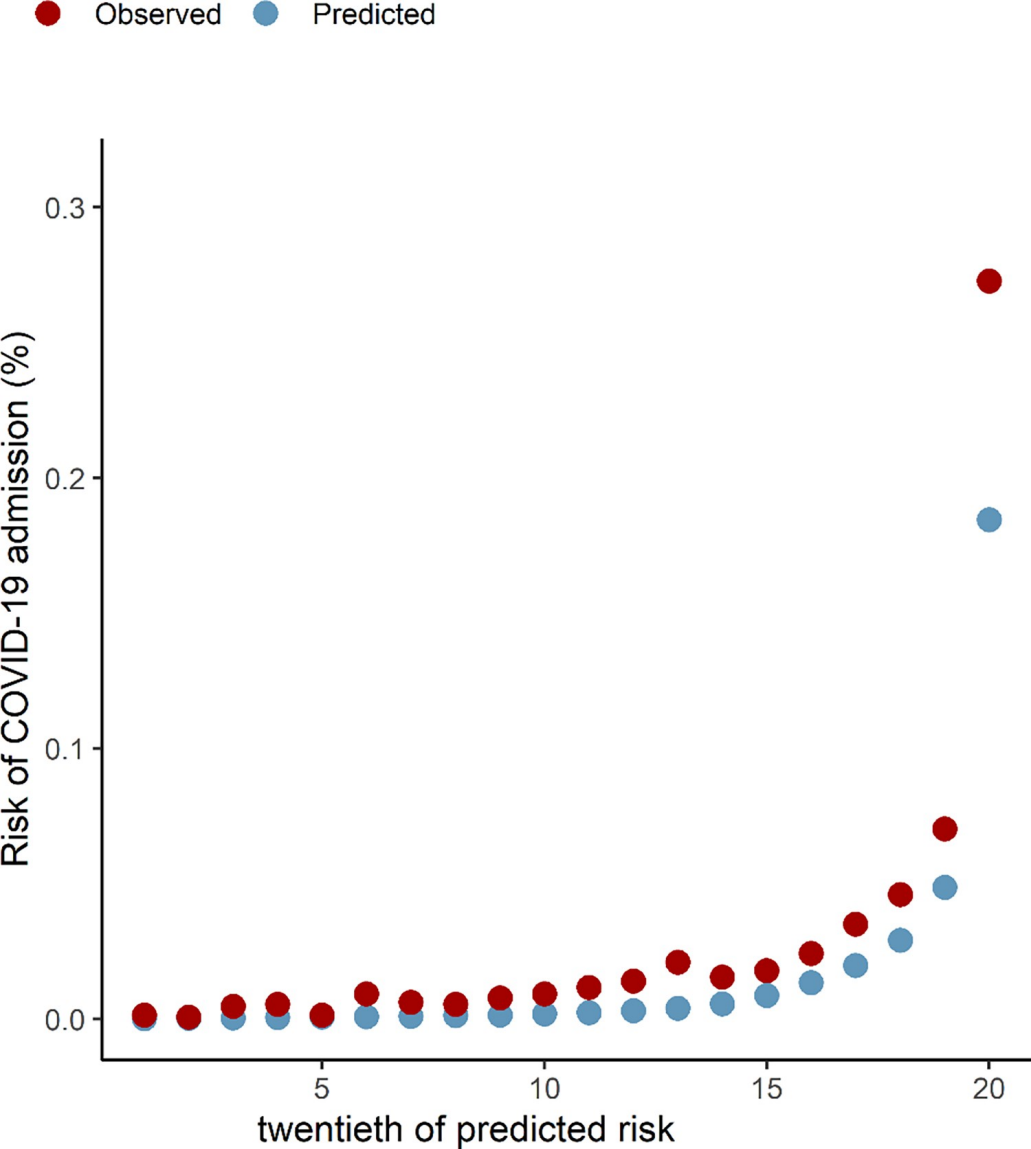
Predicted and observed risk of COVID-19 related hospital admissions.

The calibration plots in Figs 1 and 2 show that the predicted and observed risks of COVID-19-related death and hospitalisation were similar, demonstrating that the algorithms were well calibrated. However, there was slight over-prediction in the highest risk group for COVID-19-related deaths (vigintiles 18–20) and under-prediction of COVID-19-related hospitalisations, with the largest difference seen in the highest risk group.

Table 3 presents the percentage of COVID-19 related deaths at different thresholds based on centiles of predicted absolute risk. 71.4% of deaths occurred in those in the top 5% for predicted absolute risk of COVID-19 related deaths which increases to 95.2% of deaths occurring in the top 30% for predicted absolute risk of COVID-19 related deaths.

**Table 3:** Sensitivity for COVID-19 related death at different QCOVID3 thresholds of absolute risk.

Top centile	Absolute risk centile cut-off (%)	Cumulative deaths	Cumulative % deaths based on absolute risk	Cumulative % deaths based on absolute risk in English study
Top 5%	0.077	252	71.39	78.74
Top 10%	0.029	294	83.29	90.23
Top 15%	0.015	313	88.67	95.98
Top 20%	0.008	327	92.63	98.85
Top 25%	0.005	336	95.18	98.85

## Discussion

The results from this validation study demonstrate that the performance of the algorithms was good and yielded similar results to the original study in England [11]. In general, the risk algorithms showed high levels of discrimination (Harrell C statistic: ≥ 0.828 for both COVID-19 related deaths and hospital admissions) and good calibration. Improved precision in the Welsh data was shown in predicting risk of COVID-19 related death and hospitalisation in individuals who received one dose of the vaccine, and conversely lower precision was observed for risk of COVID-19 related death and hospitalisation in individuals who received two doses of the vaccine. Compared to individuals who received one vaccine dose, performance metrics were lower in both studies for those who received two doses of vaccine. The Welsh performance metrics yielded poorer results in comparison for those with two doses, but also there was a lower proportion of individuals who received two doses included, 55.6% compared to 75.7% in the English study. Individuals were followed up from 14 days after each dose of vaccine, therefore, anyone who received a first dose in the last two weeks of the study period could not be included or their second dose could not be included due to insufficient follow up time for calculating the outcomes. Additionally, as stated in the original study, there were small numbers for events occurring after second vaccination [11]. Therefore, the predictor variables for outcomes and developing the algorithm mostly came from individuals who only received one vaccine dose. This group of people are a different group from those who receive two vaccine doses. Those who received two doses early during the initial vaccination programme will have been prioritised due to occupational risk (healthcare workers) or higher risk of severe COVID-19 related outcomes [29].

We found higher observed to predicted risks for hospitalisation and lower observed to predicted risk of death in the highest risk groups in Wales (Figs 1 and 2). For COVID-19-related hospital admissions, both studies demonstrated similar trends of observed versus predicted risk, with observed admissions higher than predicted in the Welsh data in general compared to England. Overall, there was a slightly larger proportion of COVID-19 related hospital admission in the Welsh study (0.05%) compared to the English study (0.03%). Use of SAIL data allowed linkage using a demographic spine with follow-up across primary and secondary healthcare data and mortality data, and incorporates all COVID-19-related hospitalisations, including any in-hospital infections, as well as all emergency admissions within 14 days of a positive COVID-19 RT-PCR test result. This could explain the differences between the two studies and be a reason for increased observed risk in the highest risk group in Wales. Those at highest risk will have increased healthcare utilisation for any underlying conditions and were likely to partake in increased COVID-19 testing during the study period.

Proportionally, there were similar number of COVID-19 related deaths in the Welsh (0.02%) and English study (0.03%) and this is reflected in the similar calibration plots except for the highest risk group (group 20) where predicted deaths were higher than observed deaths for Wales. This could be attributed to the success of the vaccination programmes as well as protective and risk avoiding social interactions for those most at risk of serious COVID-19 outcomes. Whilst there were slight differences in the highest risk groups for risk of COVID-19 related deaths, the algorithms demonstrated 71.4% of deaths occurred in the top 5% for predicted absolute risk (Table 3) which was similar to the English study (78.7%) [11].

A recent systematic review of prediction models for severe manifestations and mortality due to COVID-19 identified 445 studies, of which 9 were rated to be low risk of bias with AUC’s ranging from 0.541 to 0.928 in populations from the UK, Ireland, Italy, Spain, Korea, US, and China [30]. The highest AUC was the creation of the original QCOVID algorithm which we had previously validated in Wales [3]. Our study focuses on using individual-level, population-wide COVID-19 risk prediction models for serious health outcomes in an adult vaccinated population, therefore, it is not possible to draw further comparisons with these earlier prediction models.

Some differences in prediction accuracy in independent populations are expected as there may be underlying differences in populations not captured by the variables included, imprecision due to relatively small numbers, and possibly differences in proportions of people treated with different modalities not captured in this study. A major strength of this study is the ability to utilise the SAIL Databank, a Trusted Research Environment, which enables population-wide, individual-level data linkage across healthcare systems to validate these pandemic predictive risk assessment algorithms. Results from this validation study in an independent population supports the findings of the QCOVID algorithm and likely to be relevant to countries with similar socio-economic conditions and health services. Understanding the demographic and clinical characteristics that are most at risk of serious health outcomes from current pandemics can be used for allocation planning for future threats and improve global equitable pandemic preparedness.

Whilst this independent study has demonstrated that the updated QCOVID algorithms fit the Welsh data well, the study includes some important limitations. As previously reported [3], the Welsh study was restricted to individuals registered to a SAIL providing general practice to derive the necessary predictor variables, therefore, results are based on 80% of the population (330/412 of all general practices in Wales). Due to SAIL’s information governance and disclosure control policies, we were unable to include information that is deemed too sensitive and therefore could not include HIV status. Some 41.6% of our cohort did not have a BMI recorded in the previous five years, therefore, missing observations were imputed. OPCS codes in hospital admissions data were used to define chemotherapy status with anyone with a record of receiving chemotherapy is assigned the coefficients for the middle severity chemotherapy group.

Also, this study replicates the original English study and so has similar stated limitations such as a relatively short follow-up, a partially vaccinated population including the Oxford-AstraZeneca or Pfizer-BioNTech COVID-19 vaccinations only, and small numbers of events in some subgroups. Consequently, it was not possible to calculate metrics by ethnic groups, or for narrowly defined age groups. Additionally, the study does not account for the interval between completion of the first and second vaccination, any changes that may have occurred in COVID-19 transmission rate within the study follow-up that might have impacted the prediction model temporally, or the different emerging variants during the study period [11]. Finally, whilst many risk factors for serious COVID-19 related outcomes have been included, additional risk factors such as occupational exposure to infection are not accounted for in this model.

## Conclusion

This study presents an independent external validation of the updated QCOVID3 risk algorithms in the adult vaccinated Welsh population and has shown that the algorithms are valid for use in the Welsh population, and applicable on a population independent of the original study, which has not been previously reported. This study provides further evidence that the QCOVID3 algorithms can help inform public health risk management on the ongoing surveillance and intervention to manage COVID-19 related risks following vaccination. The outputs from the QCOVID algorithms can be used to support the prioritisation of vaccine boosters, invitation onto clinical trials, personalised interventions for prevention of patient care with both clinicians and patients being able to calculate their own risk through the online QCOVID calculator, and support allocation planning for possible future pandemics and improve global preparedness [31].

## Supporting information

S1 Checklist STROBE statement—checklist of items that should be included in reports of observational studies. (DOCX)

S1 Fig Consort diagram of study participant inclusion. (TIF)

## Abbreviations

SAIL: Secure Anonymised Information Linkage
NERVTAG: New and Emerging Respiratory Virus Threats Advisory Group
RECORD: REporting of studies Conducted using Observational Routinely-collected health Data
WLGP: Welsh Longitudinal General Practice
PEDW: Patient Episode Database for Wales
WDDS: Wales Dispensing DataSet
CENW: Census 2011 data
BMI: Body Max Index
ICD-10: International Classification of Diseases, Tenth Revision
OPCS-4: OPCS Classification of Interventions and Procedures version 4
CKD: Chronic Kidney Disease
ONS: Office for National Statistics
ADDE: Annual District Death Extract
ADDD: Annual District Death Daily
WDSD: Welsh Demographic Service Dataset
CDDS: Consolidated Death Data Source
