# Supplementary material for: An external validation of the QCOVID3 risk prediction algorithm for risk of hospitalisation and death from COVID-19: An observational, prospective cohort study of 1.66m vaccinated adults in Wales, UK
Source: PLoS One. 2023 May 18;18(5):e0285979. doi: 10.1371/journal.pone.0285979 (PMC10194890; doi:10.1371/journal.pone.0285979)
Supplement: S1 Checklist — (DOCX) [file pone.0285979.s001.docx]

STROBE Statement—checklist of items that should be included in reports of observational studies

|  | Item No. | Recommendation | Page  No. | Relevant text from manuscript |
| --- | --- | --- | --- | --- |
| **Title and abstract** | 1 | (*a*) Indicate the study’s design with a commonly used term in the title or the abstract | 1 | An external validation of the QCOVID3 risk prediction algorithm for risk of hospitalisation and death from COVID-19: **an observational, prospective cohort study** of 1.66m vaccinated adults in Wales, UK. |
|  |  | (*b*) Provide in the abstract an informative and balanced summary of what was done and what was found | 2 | Abstract contains the objectives, methods, results and conclusion sections. |
| Introduction | | | |  |
| Background/rationale | 2 | Explain the scientific background and rationale for the investigation being reported | 3-4 | Following the emergence of the SARS-CoV-2 infection and at the start of the COVID-19 pandemic, there was an urgent public health need to identify individuals at highest risk of severe outcomes, in particular hospitalisation and death following infection.To support the National Health Service (NHS) and protect the most clinically vulnerable individuals, the Chief Medical Officer for England commissioned the New and Emerging Respiratory Virus Threats Advisory Group (NERVTAG), an expert committee of the Department of Health and Social Care who advise the UK government, to develop the QCOVID risk assessment algorithms for predicting risk of COVID-19 related hospital admission or death.  It is important to replicate and validate prediction algorithms in independent populations to ensure they work in an ‘out of sample’ setting particularly if they could be used clinically in this setting. It is also to inform policy development at a national scale and contribute to the planning and management of individual patient care |
| Objectives | 3 | State specific objectives, including any prespecified hypotheses | 4-5 | The aim of this particular study was to independently validate the updated published QCOVID3 risk prediction algorithms for risk of COVID-19-related deaths and hospitalisation in vaccinated adults having one or two doses of vaccination by 15th June 2021 in Wales, UK. |
| Methods | | | |  |
| Study design | 4 | Present key elements of study design early in the paper | 5 | We conducted an observational, longitudinal, cohort study of vaccinated adults living in Wales from 8^th^ December 2020, with follow-up until 15^th^ June 2021. The outcomes of interest were time to COVID-19 related death and hospitalisation. We assessed the performance of the QCOVID3 algorithms using measures of discrimination and calibration. |
| Setting | 5 | Describe the setting, locations, and relevant dates, including periods of recruitment, exposure, follow-up, and data collection | 5 | We conducted an observational, longitudinal, cohort study of vaccinated adults living in Wales from 8^th^ December 2020, with follow-up until 15^th^ June 2021. The outcomes of interest were time to COVID-19 related death and hospitalisation. We assessed the performance of the QCOVID3 algorithms using measures of discrimination and calibration. |
| Participants | 6 | (*a*) *Cohort study*—Give the eligibility criteria, and the sources and methods of selection of participants. Describe methods of follow-up  *Case-control study*—Give the eligibility criteria, and the sources and methods of case ascertainment and control selection. Give the rationale for the choice of cases and controls  *Cross-sectional study*—Give the eligibility criteria, and the sources and methods of selection of participants | 5-6 | We defined the population of interest as vaccinated adults living in Wales on 8^th^ December 2020 with follow-up until 15^th^ June 2021. Individuals included were aged between 19 and 100 on the 8^th^ December 2020, registered with a SAIL-providing general practice (86% of Welsh general practices), and who had received one or two vaccinations of Oxford-AstraZeneca or Pfizer-BioNTech within the study period. Follow-up started from 14 days after receiving each vaccine dose until they had the outcome of interest (COVID-19-related death or hospitalisation), died, migrated out of Wales, or until the end of the study period. Individuals who were vaccinated within 14 days of the study end date were not included due to insufficient follow up time. Individuals who only received one dose of the vaccine during the study period were followed up from 14 days post vaccination until the outcome of interest, death, migration out of Wales, or until the end of the study period. Individuals who received two doses of vaccination were, followed up over two time periods. For the first period, individuals were followed up from 14 days post first vaccination until 14 days after their second vaccination. For the second period, individuals were followed up from 14 days post second vaccination until outcome of interest, death, migration out of Wales, or until the end of the study period. |
|  |  | (*b*) *Cohort study*—For matched studies, give matching criteria and number of exposed and unexposed  *Case-control study*—For matched studies, give matching criteria and the number of controls per case |  |  |
| Variables | 7 | Clearly define all outcomes, exposures, predictors, potential confounders, and effect modifiers. Give diagnostic criteria, if applicable | 6-9 | The “Outcome of interest” and “predictor variables” sub-sections contain the definitions for all outcome measures and how the predictor variables are defined. |
| Data sources/ measurement | 8* | For each variable of interest, give sources of data and details of methods of assessment (measurement). Describe comparability of assessment methods if there is more than one group | *5-9* | The “Data sources “Outcome of interest”, and “predictor variables” sub-sections and Box 1 contain the information on what data sources were used in the study and how the variables are defined and measured. |
| Bias | 9 | Describe any efforts to address potential sources of bias | 6-7 | “Follow-up started from 14 days after receiving each vaccine dose until they had the outcome of interest (COVID-19-related death or hospitalisation), died, migrated out of Wales, or until the end of the study period. Individuals who were vaccinated within 14 days of the study end date were not included due to insufficient follow up time. Individuals who only received one dose of the vaccine during the study period were followed up from 14 days post vaccination until the outcome of interest, death, migration out of Wales, or until the end of the study period. Individuals who received two doses of vaccination were, followed up over two time periods. For the first period, individuals were followed up from 14 days post first vaccination until 14 days after their second vaccination. For the second period, individuals were followed up from 14 days post second vaccination until outcome of interest, death, migration out of Wales, or until the end of the study period.”  “We utilised a combination of ADDE, ADDD, WDSD and CDDS to identify all deaths of Welsh residents, inclusive of in-hospital and out of hospital deaths.”  “COVID-19-related hospital admission were included if they contained U07.1 or U07.2 ICD10 codes, or, any emergency admission within 14 days following a positive polymerase chain reaction (RT-PCR) COVID-19 test result. Individuals who had a COVID-19 hospitalisation prior to the study start date were not included in the hospital analysis.” |
| Study size | 10 | Explain how the study size was arrived at | 6 | Supplementary figure 1 contains flow chart for final study size |

Continued on next page

| Quantitative variables | 11 | Explain how quantitative variables were handled in the analyses. If applicable, describe which groupings were chosen and why | 7-10 | The “Predictor variables”, “Box 1”, and “Algorithm validation” sections of the paper detail how each of the variables for running the prediction models were defined and created as well as reference to the original studies and published algorithms. |
| --- | --- | --- | --- | --- |
| Statistical methods | 12 | (*a*) Describe all statistical methods, including those used to control for confounding | 10 | The “Algorithm validation” section explains the statistical models and performance metrics used to validate the prediction models. |
|  |  | (*b*) Describe any methods used to examine subgroups and interactions | 10 | “Performance metrics were calculated to validate the QCOVID3 predicted risk of COVID-19 related hospitalisation and death. R² values, D statistic, and Harrell’s C statistic with corresponding 95% intervals were calculated for the total cohort and by age, sex, and vaccination number”. |
|  |  | (*c*) Explain how missing data were addressed | 9 | “For body mass index (BMI), the latest BMI measurement within 5 years was used. BMI records outside this time period and BMIs <15 and >47 were set to missing, with the mean BMI replacing missing values.” |
|  |  | (*d*) *Cohort study*—If applicable, explain how loss to follow-up was addressed  *Case-control study*—If applicable, explain how matching of cases and controls was addressed  *Cross-sectional study*—If applicable, describe analytical methods taking account of sampling strategy | 6 | “Follow-up started from 14 days after receiving each vaccine dose until they had the outcome of interest (COVID-19-related death or hospitalisation), died, migrated out of Wales, or until the end of the study period. Individuals who were vaccinated within 14 days of the study end date were not included due to insufficient follow up time. Individuals who only received one dose of the vaccine during the study period were followed up from 14 days post vaccination until the outcome of interest, death, migration out of Wales, or until the end of the study period. Individuals who received two doses of vaccination were, followed up over two time periods. For the first period, individuals were followed up from 14 days post first vaccination until 14 days after their second vaccination. For the second period, individuals were followed up from 14 days post second vaccination until outcome of interest, death, migration out of Wales, or until the end of the study period.” |
|  |  | (*e*) Describe any sensitivity analyses |  | N/A |
| Results | | | | |
| Participants | 13* | (a) Report numbers of individuals at each stage of study—eg numbers potentially eligible, examined for eligibility, confirmed eligible, included in the study, completing follow-up, and analysed | 6 | Supplementary figure 1 contains flow chart for final study size |
|  |  | (b) Give reasons for non-participation at each stage | 6 | Supplementary figure 1 contains flow chart for final study size |
|  |  | (c) Consider use of a flow diagram | 6 | Supplementary figure 1 contains flow chart for final study size |
| Descriptive data | 14* | (a) Give characteristics of study participants (eg demographic, clinical, social) and information on exposures and potential confounders | 12-14 | Table 1: Demographic and clinical characteristics for the total cohort and those who died or were admitted to hospital with COVID-19. |
|  |  | (b) Indicate number of participants with missing data for each variable of interest | 9 | “For body mass index (BMI), the latest BMI measurement within 5 years was used. BMI records outside this time period and BMIs <15 and >47 were set to missing, with the mean BMI replacing missing values.” |
|  |  | (c) *Cohort study*—Summarise follow-up time (eg, average and total amount) | 11 | “Median follow-up time was 60 days (interquartile range 41- 76) after the first dose and 48 (22-77) days after the second dose.” |
| Outcome data | 15* | *Cohort study*—Report numbers of outcome events or summary measures over time | 12-14 | Table 1: Demographic and clinical characteristics for the total cohort and those who died or were admitted to hospital with COVID-19. |
|  |  | *Case-control study—*Report numbers in each exposure category, or summary measures of exposure |  |  |
|  |  | *Cross-sectional study—*Report numbers of outcome events or summary measures |  |  |
| Main results | 16 | (*a*) Give unadjusted estimates and, if applicable, confounder-adjusted estimates and their precision (eg, 95% confidence interval). Make clear which confounders were adjusted for and why they were included | 15,17 | Table 2. Performance of the QCOVID3 algorithm to predict risk of COVID-19 related death and hospitalisation for the total cohort and by age, sex, and vaccination dose (95% CI).  Fig 1. Predicted and observed risk of COVID-19 related deaths  Fig 2. Predicted and observed risk of COVID-19 related hospital admissions  Table 3. Sensitivity for COVID-19 related death at different QCOVID3 thresholds of absolute risk |
|  |  | (*b*) Report category boundaries when continuous variables were categorized |  | Main analysis focuses on validating risk prediction models for COVID-19 deaths and hospitalisation. Outputs include the following performance metrics: R-squared statistics, D statistics and Harrell’s C statistic by vaccine dose, age, and gender as part of a UK validation study. |
|  |  | (*c*) If relevant, consider translating estimates of relative risk into absolute risk for a meaningful time period | 17 | N/A – Table 3 contains sensitivity for COVID-19 related death at different QCOVID3 thresholds of absolute risk |

Continued on next page

| Other analyses | 17 | Report other analyses done—eg analyses of subgroups and interactions, and sensitivity analyses |  | N/A |
| --- | --- | --- | --- | --- |
| Discussion | | | | |
| Key results | 18 | Summarise key results with reference to study objectives | 18-20 | Discussion section contains the summary of the key findings from this validation study.  For example, “The results from this validation study demonstrate that the performance of the algorithms was good and yielded similar results to the original study in England [11]. In general, the risk algorithms showed high levels of discrimination (Harrell C statistic: ≥ 0.828 for both COVID-19 related deaths and hospital admissions) and good calibration. Improved precision in the Welsh data was shown in predicting risk of COVID-19 related death and hospitalisation in individuals who received one dose of the vaccine, and conversely less precision was observed for risk of COVID-19 related death and hospitalisation in individuals who received two doses of the vaccine.” |
| Limitations | 19 | Discuss limitations of the study, taking into account sources of potential bias or imprecision. Discuss both direction and magnitude of any potential bias | 20 | “Whilst this independent study has demonstrated that the updated QCOVID algorithms fit the Welsh data well, the study includes some important limitations. As previously reported [3], the Welsh study was restricted to individuals registered to a SAIL providing general practice to derive the necessary predictor variables, therefore, results are based on 80% of the population (330/412 of all general practices in Wales). Due to SAIL’s information governance and disclosure control policies, we were unable to include information that is deemed too sensitive and therefore could not include HIV status. Some 41.6% of our cohort did not have a BMI recorded in the previous five years, therefore, missing observations were imputed. OPCS codes in hospital admissions data were used to define chemotherapy status with anyone with a record of receiving chemotherapy is assigned the coefficients for the middle severity chemotherapy group.  Also, this study replicates the original English study and so has similar stated limitations such as a relatively short follow-up, a partially vaccinated population, and small numbers of events in some subgroups. Consequently, it was not possible to calculate metrics by ethnic groups, or for narrowly defined age groups. Finally, the study does not account for different emerging variants during the study period” |
| Interpretation | 20 | Give a cautious overall interpretation of results considering objectives, limitations, multiplicity of analyses, results from similar studies, and other relevant evidence | 20-21 | “This study presents an independent external validation of the updated QCOVID3 risk algorithms in the adult vaccinated Welsh population and has shown that the algorithms are valid for use in the Welsh population, and applicable on a population independent of the original study, which has not been previously reported. This study provides further evidence that the QCOVID3 algorithms can help inform public health risk management on the ongoing surveillance and intervention to manage COVID-19 related risks following vaccination. The outputs from the QCOVID algorithms can be used to support the prioritisation of vaccine boosters, invitation onto clinical trials, and personalised interventions for prevention of patient care with both clinicians and patients being able to calculate their own risk through the online QCOVID calculator” |
| Generalisability | 21 | Discuss the generalisability (external validity) of the study results | 17-21 | This paper is an independent validation of the published QCOVID algorithm and validity and comparison with published studies is discussed throughout the discussion section. |
| Other information | |  | | |
| Funding | 22 | Give the source of funding and the role of the funders for the present study and, if applicable, for the original study on which the present article is based | Included in a supplementary file | “This work was supported by the Con-COV team funded by the Medical Research Council (grant number: MR/V028367/1). This work was supported by Health Data Research UK, which receives its funding from HDR UK Ltd (HDR-9006) and the Medical Research Council (MR/ S027750/1). HDR UK Ltd is funded by the UK Medical Research Council, Engineering and Physical Sciences Research Council, Economic and Social Research Council, Department of Health and Social Care (England), Chief Scientist Office of the Scottish Government Health and Social Care Directorates, Health and Social Care Research and Development Division (Welsh Government), Public Health Agency (Northern Ireland), British Heart Foundation (BHF) and the Wellcome Trust. This work was supported by the ADR Wales programme of work. The ADR Wales programme of work is aligned to the priority themes as identified in the Welsh Government’s national strategy: Prosperity for All. ADR Wales brings together data science experts at Swansea University Medical School, staff from the Wales Institute of Social and Economic Research, Data and Methods (WISERD) at Cardiff University and specialist teams within the Welsh Government to develop new evidence which supports Prosperity for All by using the SAIL Databank at Swansea University, to link and analyse anonymised data. ADR Wales is part of the Economic and Social Research Council (part of UK Research and Innovation) funded ADR UK (grant ES/S007393/1). This work was supported by the Wales COVID-19 Evidence Centre, funded by Health and Care Research Wales.  The original development and validation of the QCOVID algorithms were funded by the National Institute for Health Research (NIHR) following a commission by the Chief Medical Officer for England. QResearch was supported by funds from the John Fell Oxford University Press Research Fund, grants from Cancer Research UK (grant C5255/A18085), through the Cancer Research UK Oxford Centre, and, grants from the Oxford Wellcome Institutional StrategicSupport Fund (204826/Z/16/Z), during the conduct of the study. KK is supported by the National Institute for Health Research (NIHR) Applied Research Collaboration East Midlands (ARC EM) and the NIHR Leicester Biomedical Research Centre (BRC).” |

*Give information separately for cases and controls in case-control studies and, if applicable, for exposed and unexposed groups in cohort and cross-sectional studies.

**Note:** An Explanation and Elaboration article discusses each checklist item and gives methodological background and published examples of transparent reporting. The STROBE checklist is best used in conjunction with this article (freely available on the Web sites of PLoS Medicine at http://www.plosmedicine.org/, Annals of Internal Medicine at http://www.annals.org/, and Epidemiology at http://www.epidem.com/). Information on the STROBE Initiative is available at www.strobe-statement.org.
