# Supplementary figures and images for: An external validation of the QCOVID3 risk prediction algorithm for risk of hospitalisation and death from COVID-19: An observational, prospective cohort study of 1.66m vaccinated adults in Wales, UK
Source: PLoS One. 2023 May 18;18(5):e0285979. doi: 10.1371/journal.pone.0285979 (PMC10194890; doi:10.1371/journal.pone.0285979)

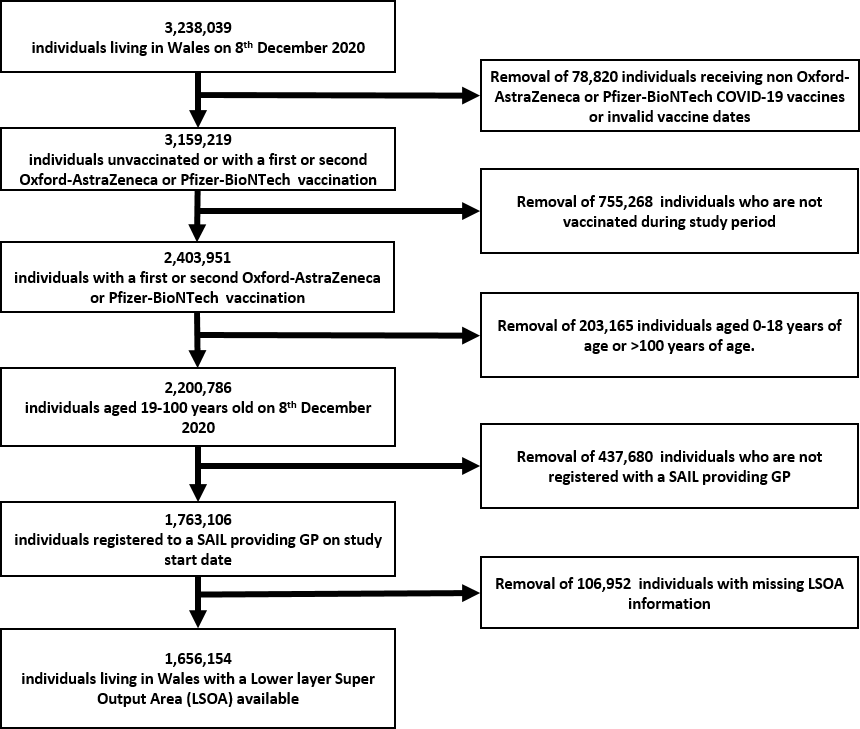

Supplement: S1 Fig — (TIF) [file pone.0285979.s002.tif]
